# Management of Low-Grade Ductal Carcinoma In Situ in a Supernumerary Breast: A Case Report

**DOI:** 10.7759/cureus.94169

**Published:** 2025-10-09

**Authors:** Alicia Bui, Jacob F Oyeniyi, Bailey A Loving, Jean Llenos, James Fontanesi

**Affiliations:** 1 Medicine, Oakland University William Beaumont School of Medicine, Royal Oak, USA; 2 Radiation Oncology, Corewell Health William Beaumont University Hospital, Royal Oak, USA; 3 Pathology, Corewell Health William Beaumont University Hospital, Royal Oak, USA; 4 Radiation Oncology, Corewell Health Farmington Hills Hospital, Farmington Hills, USA

**Keywords:** ductal carcinoma in situ (dcis), ectopic breast cancer, external beam radiation, multidisciplinary cancer care, supernumerary breast tissue

## Abstract

Supernumerary breast tissue arising from incomplete regression of the mammary ridges is rare. This ectopic tissue is affected by the same hormonal influences as a normal breast and can lead to both benign and malignant changes. We describe a case of a female with ductal carcinoma in situ (DCIS) in ectopic breast tissue located below the left inframammary fold. The lesion was surgically excised and treated with adjuvant external beam radiation to the tumor bed. Ectopic breast carcinoma is a rare condition and is often undetected by screening mammography due to its unusual location. Management should involve a multidisciplinary team and adhere to established protocols for breast cancer treatment. We discuss the role of radiotherapy in the treatment of ectopic breast carcinoma.

## Introduction

The presence of supernumerary breast tissue is a rare condition that results from remnants of the mammary ridges that fail to regress during embryologic development [1]. Breast development begins during the 4th week of pregnancy when mammary glands arise as bilateral ectodermal thickenings that extend from the axilla to the groin. By the 5th week, this ridge regresses, except in the pectoral region, where it later forms breasts [2]. Incomplete regression results in the development of ectopic breast tissue, manifesting as polymastia or polythelia along the “milk line”. Supernumerary breast tissue can respond to hormonal stimulation and is vulnerable to the same pathologic and physiologic changes that affect orthotopic breast tissue [1,2].

The prevalence of ectopic breast tissue is low, occurring in 0.2-6% of women, with the axilla being the most common site [3]. Carcinoma arising in ectopic breast tissue is exceedingly rare, accounting for 0.2-0.6% of all breast cancer cases [4]. The management of supernumerary breast cancer should adhere to the same approach used for standard breast cancer, including clinical examination, mammography, ultrasound, and either fine needle aspiration cytology or core needle biopsy [5]. Screening mammograms can miss these lesions due to their atypical location, making the diagnosis more challenging [6,7].

Given the rarity of supernumerary breast cancer, there is no consensus or clear guideline on its management. Therapeutic options for ectopic breast malignancies are based on standard breast cancer treatment for breast cancer arising in the orthotopic/normally located breast, involving a combination of surgical excision, radiation therapy, and systemic therapy tailored to the tumor’s characteristics [5,8,9]. Many women with DCIS choose breast-conserving treatment, with the primary decision being whether to include radiation therapy after lumpectomy. Adjuvant radiation therapy after breast-conserving surgery for DCIS is a category 1 recommendation in the National Comprehensive Cancer Network (NCCN) breast cancer guideline, as it reduces local recurrence by 50-70%, half of which recur as invasive disease [9-11]. A review of the literature offers no formal guidance on radiotherapy dose and fractionation in the context of ectopic breast carcinoma [12]. We describe a case of ductal carcinoma in situ in supernumerary breast tissue treated with surgical excision and external beam radiation.

## Case presentation

**Figure 1:**
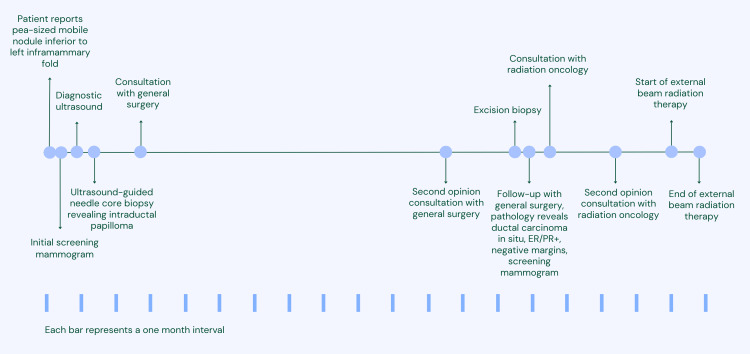
Timeline of patient's care episode. ER: estrogen receptor; PR: progesterone receptor

A woman in her 60s presented with a painless, recently identified, pea-sized mobile nodule on the chest wall ~5 cm inferior to the left inframammary fold in the midclavicular line. The patient had no detectable breast masses, and her prior yearly mammograms had consistently shown no suspicious lesions. She had never undergone a breast biopsy, was postmenopausal, and had not used hormone replacement therapy. The patient has a family history of ductal carcinoma in situ in a first-degree relative, which was treated with surgery, radiotherapy and tamoxifen. She had no other contributory history. Figure 1 shows a graphical representation of the patient’s timeline of events.

**Figure 2:**
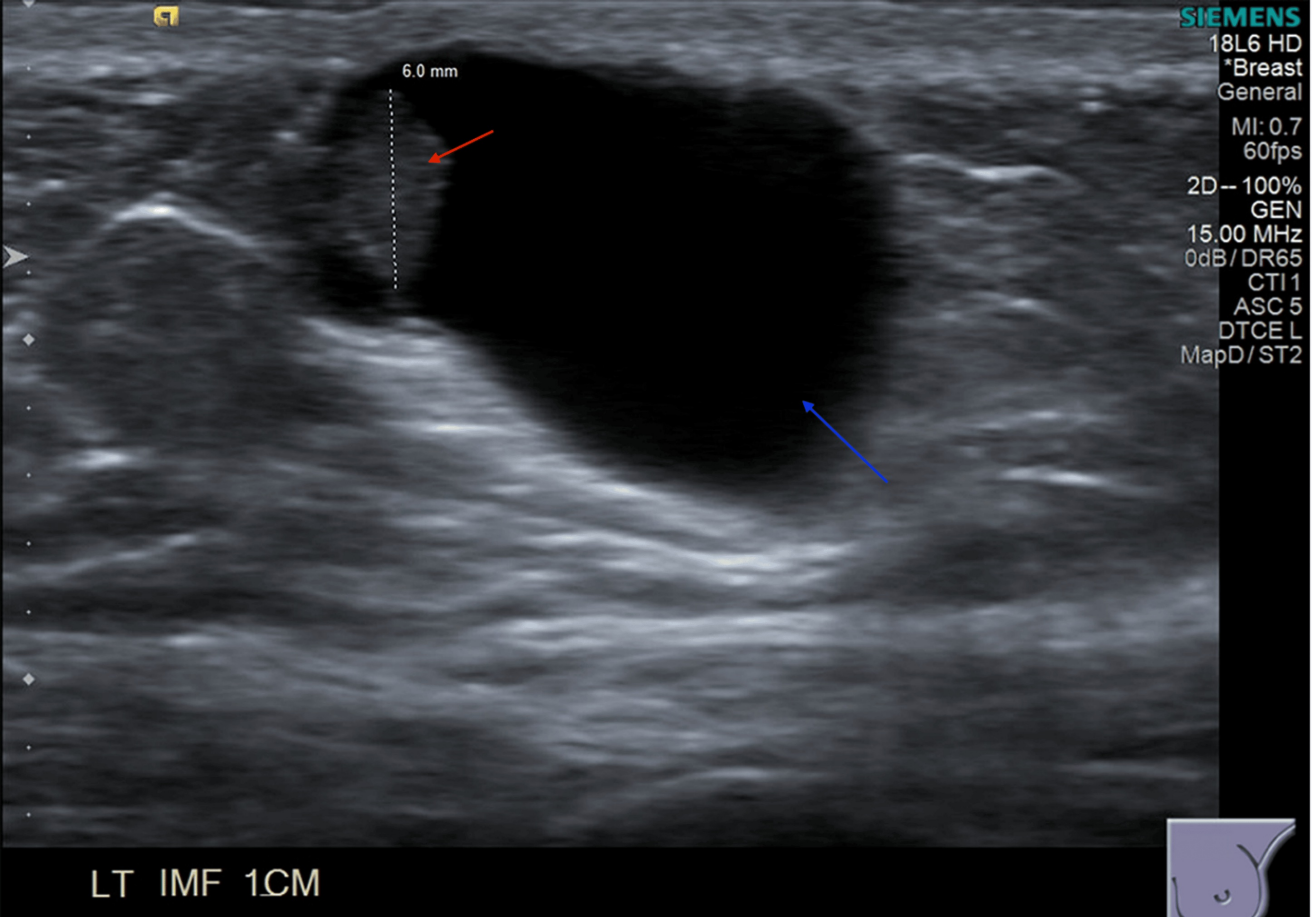
Diagnostic ultrasound image of left inframammary lesion depicting a 1.9 x 1.3 x 1.1 cm cystic lesion (blue arrow) with mural nodule measuring 6 mm (red arrow).

**Figure 3:**
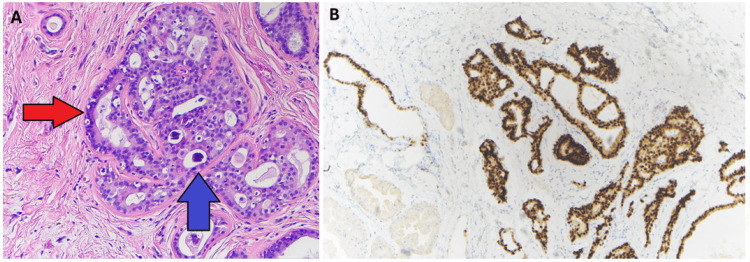
Histopathology (A) 40x magnification images of a hematoxylin & eosin (HE) section from a left supernumerary breast specimen. Slide is from a cystic space lined with papillary projections, with microscopy demonstrating multiple nests of tumor cells with nuclear pleomorphism (red arrow) and loss of nuclear polarity, along with some microcalcifications (blue arrow). (B) Cells stained positively for estrogen receptor.

Workup with screening mammogram showed no evidence of malignancy (Breast Imaging Reporting and Data System (BI-RADS) 1). Diagnostic ultrasound revealed a 1.9 x 1.3 x 1.1 cm cystic lesion with a mural nodule measuring 6 mm that corresponded with the area of palpable concern and is depicted in Figure 2. An ultrasound-guided core biopsy of the mass was performed, and histopathology subsequently identified intraductal papilloma without evidence of atypia or malignancy. After biopsy, the mass decreased in size, but the patient noted regrowth 4 months later. After extensive discussion with the patient, an excisional biopsy was performed, and histopathology revealed low-grade ductal carcinoma in situ with microcalcifications measuring at least 5 mm (Figure 3). All margins were negative, with the distance to the closest margin measuring 1 mm. The immunohistochemistry study showed positive estrogen (97%) and progesterone (94%) receptors. A screening mammogram at this time was benign BI-RADS 2.

**Figure 4:**
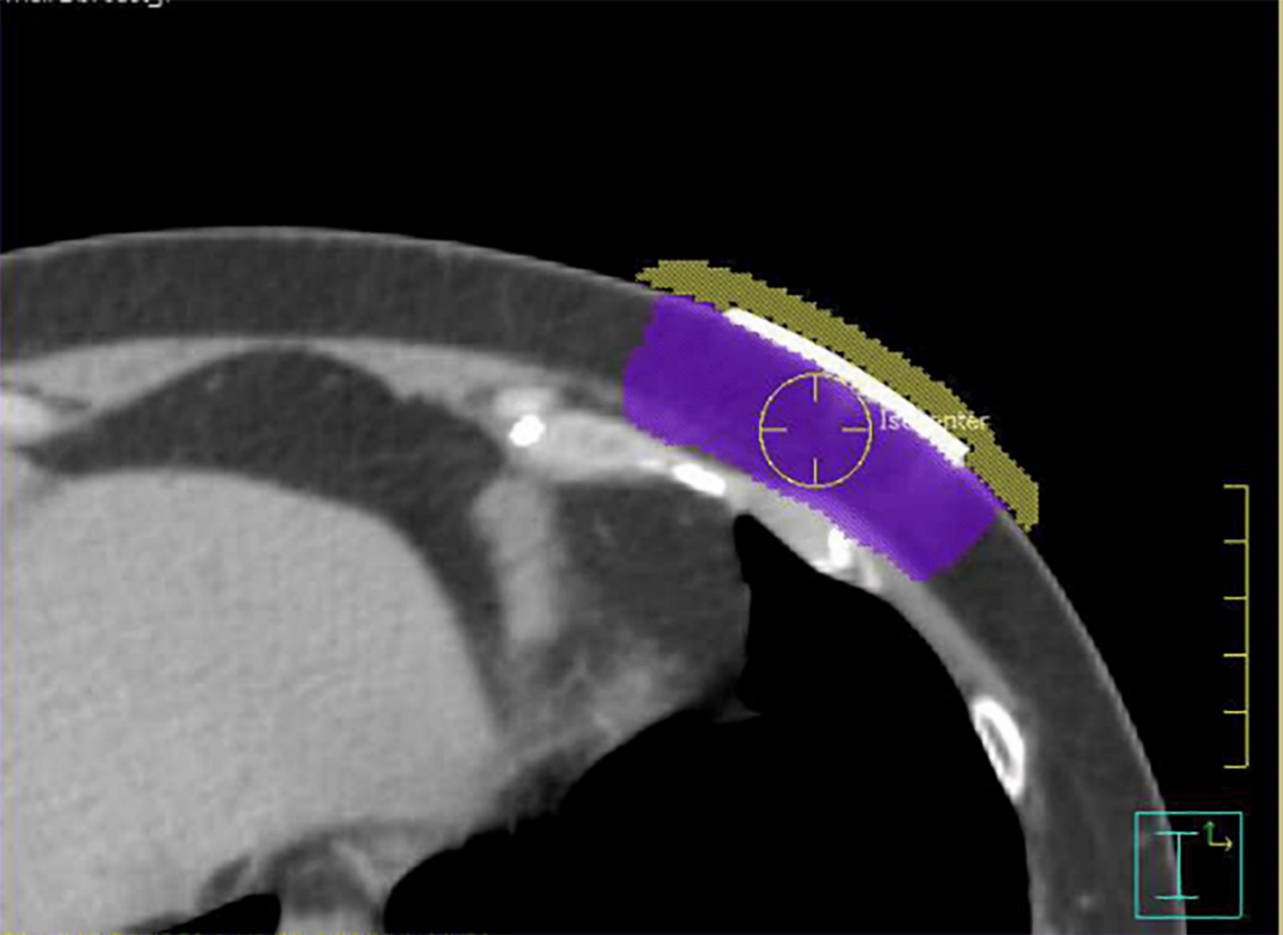
Axial CT slice showing bolus (yellow) and PTV in the left chest wall (purple). PTV: planning target volume

A multidisciplinary board, including breast surgical oncology, medical oncology, radiation oncology, pathology, and radiology, amongst others, recommended adjuvant radiation therapy and consideration of endocrine therapy without further surgery. DCISionRT® testing (PreludeDx, Laguna Hills, USA) was inconclusive due to an insufficient sample. External beam radiation therapy was completed with 16 fractions of 2.66 Gy for a total dose of 42.56 Gy over 21 days. The planning target volume (PTV) was the tumor bed + 1 cm radially in the left chest wall. The radiation was administered using unopposed tangents of 6 MeV photons with a 0.5 cm bolus using a 3D conformal radiation therapy technique (Figure 4).

At the time of treatment completion, the patient experienced mild skin erythema over the treatment area, which had completely resolved by her 6-month posttreatment follow-up. Clinical examination and her screening mammogram at that time were negative. Of note, hormonal therapy was offered by the medical oncologist, but the patient declined due to concerns about side effects.

## Discussion

Supernumerary breast tissue, although rare, presents unique challenges and considerations, particularly when malignancies such as DCIS arise within this ectopic tissue. The differential diagnosis of a subcutaneous nodule below the breast includes benign entities such as lipoma, epidermal inclusion cyst, sebaceous cyst, and ectopic breast tissue, as well as malignant lesions like metastatic deposits and soft tissue sarcomas. Ectopic breast tissue may be distinguished clinically by its alignment along the embryonic milk line and histologically by its resemblance to orthotopic breast tissue. Identifying supernumerary breast lesions is challenging due to their ectopic location, which is often missed on screening mammography [6,7]. Therefore, some authors recommended that ectopic breast tissue be included in routine screening procedures [5].

Although ectopic breast tissue was previously thought to be at higher risk for malignant transformation, Marshall et al. concluded that there is no increased incidence of cancer in supernumerary breast tissue compared to normal breast tissue [2]. Ectopic breast malignancies were historically managed with an aggressive surgical approach; however, local wide excision with adjuvant radiotherapy in accordance with standard breast cancer treatment is currently preferred [5,8]. Nevertheless, comprehensive guidelines for the use of radiotherapy in the treatment of ectopic breast cancer remain sparse in the literature. Hallam et al. report a case of grade 2 estrogen and progesterone receptor-positive invasive ductal carcinoma treated with wide local excision, adjuvant radiotherapy with 9 MeV electrons to a dose of 40 Gy in 15 fractions over 21 days, and tamoxifen [11]. Careful consideration was made to treat the tumor bed alone to minimize toxicity and reduce late cosmetic effects. This regimen resulted in no evidence of recurrence and an excellent cosmesis at her 6-month follow-up. We delivered a dose of 42.5 Gy in 16 fractions, which is a standard dose that aligns with the National Comprehensive Cancer Network guidelines [9,13].

This case highlights the limitations of screening mammography in patients with supernumerary breasts. On the other hand, MRI is not a routine screening method for breast cancer, except for patients deemed high-risk. There is no established evidence suggesting that individuals with supernumerary breast tissue are at a higher baseline risk of breast cancer. Thus, a screening MRI would not typically be recommended. There should be a low threshold for evaluation of a newly noticed or changing supernumerary breast tissue, typically with a diagnostic ultrasound. A diagnostic MRI may be useful in select cases with inconclusive ultrasound or complex anatomy. In our case, an MRI was not performed due to the lesion’s superficial location and clear sonographic visualization.

It is difficult to determine the prognosis for patients with ectopic breast carcinoma due to the small number of reported cases and limited follow-up data. Lymph node and distant metastases have been reported at the time of initial diagnosis, with challenges in screening and delayed detection contributing to this presentation [14,15]. Some authors recommend prophylactic excision of supernumerary breast tissue as a preventive measure against malignancy [4]. While prospective studies on this topic are lacking due to the rarity of the condition, the concept is based on principles similar to prophylactic mastectomy in high-risk populations. Given the lack of functional necessity and potential cosmetic concerns with supernumerary breast tissue, this may be reasonable for appropriately motivated patients [4]. Evans and Guyton reviewed 90 patients and concluded that recurrence and survival were comparable between patients who received mastectomy or less extensive treatment, wide local excision, and radiation [14].

Predictive tools such as DCISionRT - a genomic assay that combines molecular and clinical data to generate a personalized risk score - may play a role in assessing the risk of recurrence and predicting the benefit of adjuvant radiation therapy following breast-conserving surgery in patients with ectopic breast DCIS. Although DCISionRT has been validated by prospective clinical studies, such as the PREDICT study, which demonstrated that it influenced radiotherapy recommendations in 38% of women with DCIS and reduced overtreatment, it has not yet been integrated into clinical guidelines or routine practice in most clinics and is typically used to identify low-risk DCIS patients for whom RT can potentially be omitted [17]. Given our patient’s inconclusive DCISionRT result, it did not alter our treatment strategy.

Standard adjuvant treatment was pursued based on multidisciplinary discussion and the patient’s preference for aggressive local control, prioritizing recurrence risk reduction in line with standard breast conservation strategies. Further investigation of the utility of DCISionRT may be warranted in scenarios where patients with ectopic breast DCIS are hesitant about radiotherapy or when the benefit of radiotherapy is uncertain.

## Conclusions

In conclusion, supernumerary breast carcinoma is a rare condition, and its diagnosis is hindered by the ectopic location of lesions, which cannot be detected by screening mammograms. The management of primary ectopic breast carcinoma should follow a multidisciplinary approach and align with standard breast cancer treatment principles. Additional reports on radiotherapy treatment plans may establish formal guidelines for dosing, fractionation, and treatment fields in ectopic breast cancer.
